# Metabolic changes associated with metformin potentiates Bcl-2 inhibitor, Venetoclax, and CDK9 inhibitor, BAY1143572 and reduces viability of lymphoma cells

**DOI:** 10.18632/oncotarget.24989

**Published:** 2018-04-20

**Authors:** Vineela Chukkapalli, Leo I. Gordon, Parameswaran Venugopal, Jeffrey A. Borgia, Reem Karmali

**Affiliations:** ^1^ Departments of Hematology, Oncology and Stem Cell Therapy, Rush University Medical Center, Chicago, IL, USA; ^2^ Division of Hematology/Oncology, Northwestern University Feinberg School of Medicine, Chicago, IL, USA; ^3^ Robert H. Lurie Comprehensive Cancer Center of Northwestern University, Chicago, IL, USA; ^4^ Departments of Pathology and Cell and Molecular Medicine, Rush University Medical Center, Chicago, IL, USA

**Keywords:** double hit lymphoma, metformin, Bcl-2 inhibitor, CDK9 inhibitor, Venetoclax

## Abstract

Metformin exerts direct anti-tumor effects by activating AMP-activated protein kinase (AMPK), a major sensor of cellular metabolism in cancer cells. This, in turn, inhibits pro-survival mTOR signaling. Metformin has also been shown to disrupt complex 1 of the mitochondrial electron transport chain. Here, we explored the lymphoma specific anti-tumor effects of metformin using Daudi (Burkitt), SUDHL-4 (germinal center diffuse large B-cell lymphoma; GC DLBCL), Jeko-1 (Mantle-cell lymphoma; MCL) and KPUM-UH1 (double hit DLBCL) cell lines. We demonstrated that metformin as a single agent, especially at high concentrations produced significant reductions in viability and proliferation only in Daudi and SUDHL-4 cell lines with associated alterations in mitochondrial oxidative and glycolytic metabolism. As bcl-2 proteins, cyclin dependent kinases (CDK) and phosphoinositol-3- kinase (PI3K) also influence mitochondrial physiology and metabolism with clear relevance to the pathogenesis of lymphoma, we investigated the potentiating effects of metformin when combined with novel agents Venetoclax (bcl-2 inhibitor), BAY-1143572 (CDK9 inhibitor) and Idelalisib (p110δ- PI3K inhibitor). Co-treating KPUM-UH1 and SUDHL-4 cells with 10 mM of metformin resulted in 1.4 fold and 8.8 fold decreases, respectively, in IC-50 values of Venetoclax. By contrast, 3-fold and 10 fold reduction in IC-50 values of BAY-1143572 in Daudi and Jeko-1 cells respectively was seen in the presence of 10 mM of metformin. No change in IC-50 value for Idelalisib was observed across cell lines. These data suggest that although metformin is not a potent single agent, targeting cancer metabolism with similar but more effective drugs in novel combination with either bcl-2 or CDK9 inhibitors warrants further exploration.

## INTRODUCTION

In 2017, it is estimated that approximately 72,240 new cases of non-Hodgkins lymphoma (NHL) will be diagnosed in the United States, making NHL the 7th most common cause of cancer mortality with an estimated 20,140 patient deaths this year alone [1]. While the addition of anti-CD20 monoclonal antibody, rituximab, to standard chemotherapy in the late 1990's was a significant step in improving survival rates in aggressive lymphoma, patient subsets with more aggressive features will typically have chemotherapy-refractory disease. As an example, patients with diffuse large B-cell lymphoma (DLBCL) who harbor a translocation at the c-*MYC* locus have less favorable rates of response to therapy and disease free survival compared to patients without the mutation, owing to the oncogenic effects of c-*MYC* [2]. Among those with the worst outcomes are patients with “double hit” lymphomas (DHL), defined by the presence of a c-*MYC* mutation in conjunction with the B cell leukemia-2 (*BCL-2*) translocation. Adding to the challenge of managing such patients is the paucity of data on how to optimally treat these very high risk patients, with current treatment strategies falling short [3]. Similarly, mantle cell lymphoma (MCL) represents another challenging lymphoma subtype, owing to an aggressive and variable disease course and biology, a high rate of relapse, and lack of a standardized treatment approach [4–6]. These subset populations are in great need of novel therapeutic strategies. Metabolic reprogramming of tumor biology represents one viable approach.

There is growing evidence tying tumor progression and chemotherapy resistance to dysregulated cellular metabolism and mitochondrial uncoupling in hematologic malignancies [7–10]. Many tumor cells adopt a metabolic phenotype characterized by high rates of glucose uptake with aerobic glycolysis; metabolic intermediates produced from glucose metabolism and increased non-oxidative ATP are channeled to tumor cell growth, a phenomenon known as the “Warburg Effect” [11, 12]. Adenosine monophosphate-activated protein kinase (AMPK), an important sensor of cellular energy that also plays a crucial role in B-cell development [13], has long been known to inhibit pro-survival mTOR signaling. More recently, it has been demonstrated that AMPK functions as a negative regulator of the Warburg Effect and suppresses tumor growth *in vivo* [14].

Metformin, an oral anti-diabetic agent, activates AMPK either via the tumor suppressor kinase LKB1 [15], or by promoting an increase in AMP:ATP ratios through modulation of mitochondrial electron transport [16, 17] with resultant inhibition of mTOR. Metformin has the added benefit of down-regulating the effects of various pro-oncogenic pathways including insulin/PI3K/Akt, and c-MYC signaling [18–20]. Observational studies have suggested that exposure to metformin improves survival in diabetic patients with various cancers including DLBCL [20–22]. Shi and colleagues first provided insight into the anti-lymphoma specific mechanism of metformin as an AMPK agonist and promoter of tumor cell autophagy when combined with an anthracycline [19]. Further, our group recently identified a metagene of interacting proteins associated with both metformin therapeutic effect and overall survival specific to DHL and double protein expressing patients [23]. However, *in vitro* data characterizing the effects of metformin on mitochondrial respiration and cellular metabolism in B-cell lymphomas remains relatively rudimentary while the differential therapeutic effects of metformin across various histologic subtypes of aggressive B-cell lymphomas and according to c-*MYC* status has yet to be explored.

The bcl-2 family of proteins, cyclin dependent kinases (CDK) and phosphoinositol-3-kinase (PI3K) are applicable to mitochondrial physiology and cellular metabolism with clear relevance to the pathogenesis and treatment resistance of lymphoma [20, 24–26]. The cooperation of these proteins with AMPK driven processes have not been well studied in lymphoma. Moreover, whether metformin can potentiate the effects of novel agents that target these alternative pathways is unknown.

Here we characterize alterations in mitochondrial respiration with metformin *in vitro* in several aggressive B-cell lymphoma cell lines. We show that metformin has differential effects as a single agent and uncover the broader effects of metformin when combined with novel targeted agents bcl-2 inhibitor Venetoclax and CDK9 inhibitor BAY-1143572. Specifically, we observe that metformin increases sensitivity to both Venetoclax and BAY-1143572 in specific cell histologies.

## RESULTS

### Cell viability of lymphoma cells is reduced by metformin in a cell-line dependent manner

**Figure 1:**
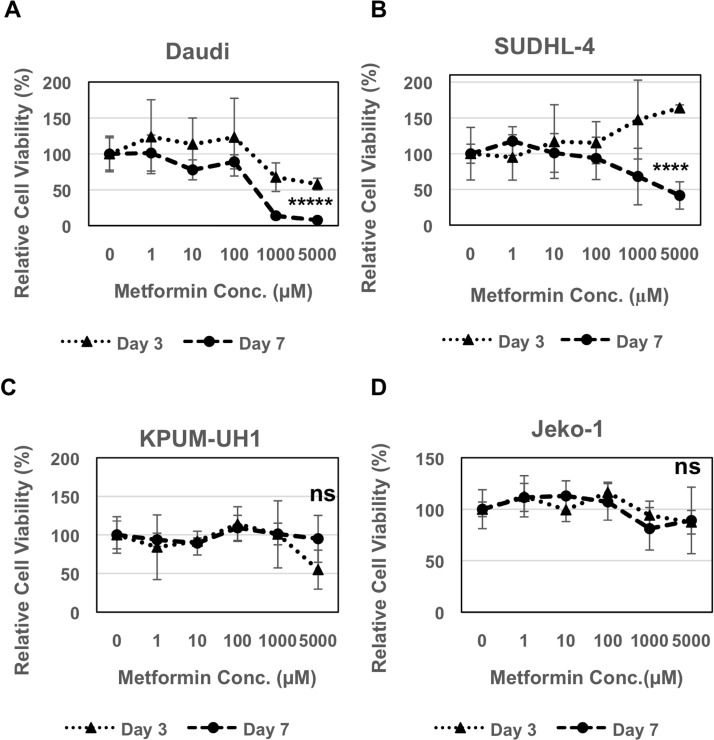
Metformin reduces viability of lymphoma cells 2000 cells (**A** Daudi, **B** SUDHL-4, **C** KPUM-UH1, **D** Jeko-1) were plated in each well of a 96 well plate and treated with different concentrations of metformin (0 μM, 1 μM, 10 μM, 100 μM, 1000 μM, 5000 μM: 5 replicates per condition). Cells were pelleted, washed once with 1X PBS and frozen at day 3 and 7. The number of cells were quantified using Hoechst 33342 DNA-binding dye after multiple freeze-thaw cycles to lyse the cells. Fluorescence reading of Hoechst 33342 at 0 μM on all days was set to 100% and was used to normalize the rest of the data. Error bars represent standard deviation between replicates. *p* values between 0 μM vs. 5000 μM on day 7 is shown. ^*****^p value < 0.0001; ^****^p value = 0.0005; ns = not significant.

Daudi, SUDHL-4, KPUM-UH1 or Jeko-1 cells were plated in each well of a 96-well plate and treated with a dose-response series of concentrations of metformin (0 −5000 μM). The number of cells at days 3 and 7 were quantified using Hoechst 33342 DNA assay. Daudi cells showed reduced viability in both a concentration and time-dependent manner. 40% reduction in viability was seen on day 3 with both 1000 μM (*p* value = 0.05) and 5000 μM (*p* value = 0.007) of metformin, whereas 80–90% reduction was seen on Day 7 (*p* value < 0.0001; Figure 1A). SUDHL-4 cells were more resistant but were still sensitive to 5000 μM of metformin on Day 7 showing 60% reduction in viability (*p* value: 0.0005; Figure 1B). On the other hand, KPUM-UH1 and Jeko-1 cells seem to be resistant to metformin treatment with no significant change in viability even at higher concentrations (Figure 1C and 1D). Taken together, these data show that metformin reduces viability in a cell-line dependent manner.

### Metformin inhibits oxidative phosphorylation in lymphoma cells

Metformin has been shown to activate AMPK and inhibit complex I of the electron transport chain. To determine the overall energetic and metabolic changes induced by metformin, especially in sensitive cells, we employed the Seahorse XF Cell Energy Phenotype Test (Agilent) as detailed in the Materials and Methods section. Both Daudi and SUDHL-4 cells were pretreated with 0 μM, 100 μM, 1 mM of metformin for 7 days and their basal oxygen consumption rate (OCR) and extra-cellular acidification rate (ECAR) measurements were taken on our Seahorse XF24 analyzer (Agilent). Cells were then stressed with the combination of 1 μM of oligomycin and 0.5 μM or 1 μM of FCCP respectively for Daudi or SUDHL-4. Stressed OCR and ECAR measurements were then taken. For additional details on the Seahorse assays please visit http://www.agilent.com/en-us/promotions/seahorse-xf-technology.

**Figure 2:**
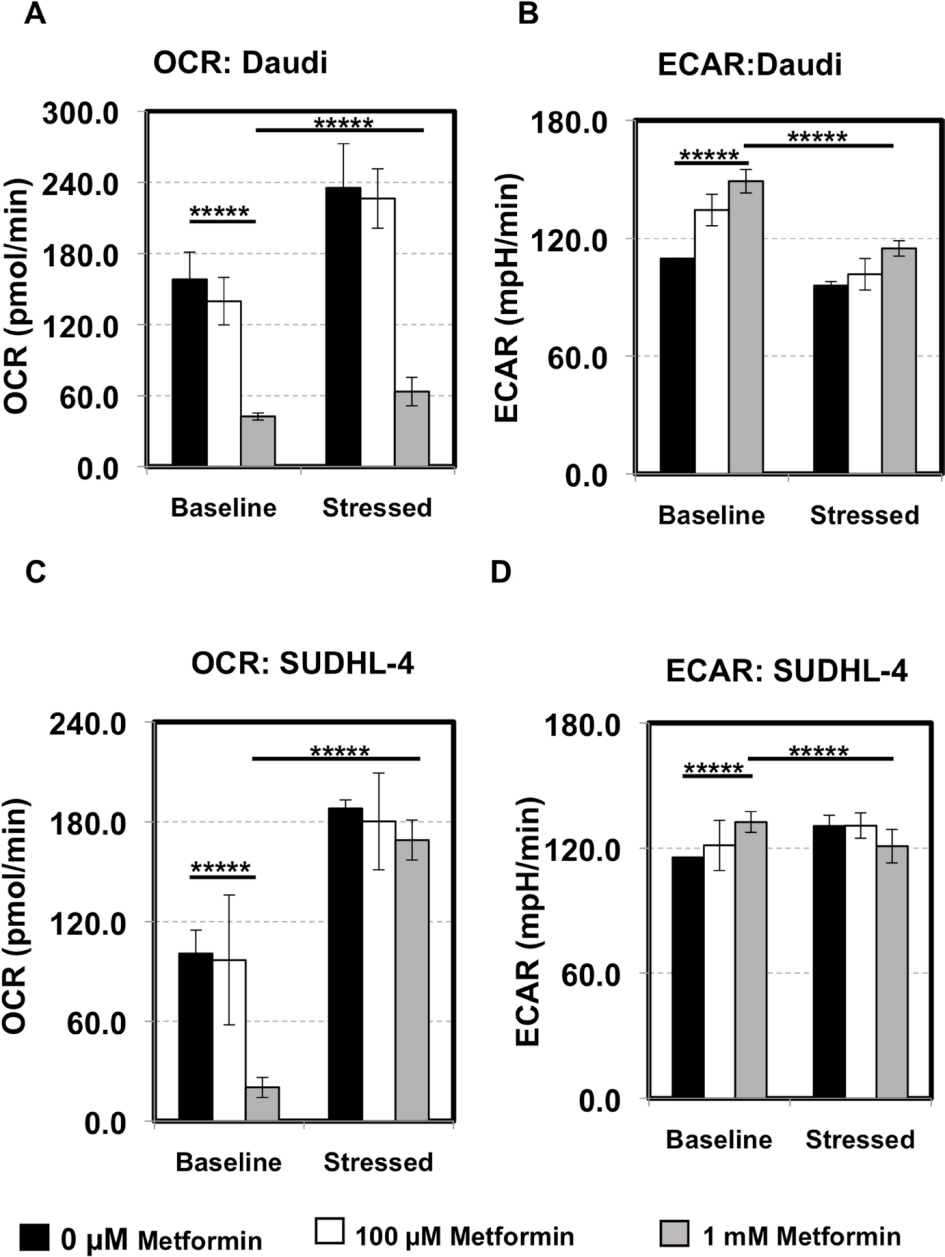
Lymphoma cells prefer oxidative phosphorylation over glycolysis Daudi cells (**A**, **B**) and SUDHL-4 (**C**, **D**) were pretreated with 0 μM (black), 100 μM (white), 1 mM (grey) of metformin for 7 days and their metabolic state was determined using the Seahorse XF Cell Energy Phenotype kit (Agilent) in triplicate. In short, after basal Oxygen Consumption Rate (OCR) and Extracellular Acidification Rate (ECAR) measurements, cells were stressed with the combination of 1 μM oligomycin and 0.5 μM FCCP for Daudi or 1 μM FCCP for SUDHL-4 and stressed OCR and ECAR measurements were taken. No metformin was present during the assay (2 hours). (A, C) OCR: The rate of decrease of oxygen concentration in the assay medium. OCR is a measure of the rate of mitochondrial respiration of the cells. (B, D) ECAR: The rate of increase in proton concentration [or decrease in pH] in the assay medium. ECAR is a measure of the rate of glycolysis of the cells. Baseline: OCR and ECAR of cells at starting assay conditions (specifically, in the presence of non-limiting quantity of substrates). Stressed: OCR and ECAR of cells under an induced energy demand (specifically, in the presence of stressor compounds). *p* values for basal OCR/ECAR at 0 μM vs 1 mM of metformin and basal OCR/ECAR vs. stressed OCR/ECAR at 1 mM metformin is shown. ^*****^p value < 0.0001.

As seen in Figure 2A and 2C, pretreating unstressed Daudi and SUDHL-4 cells particularly with 1 mM of metformin for 7 days reduced basal OCR significantly indicating that oxidative phosphorylation (or mitochondrial respiration) is affected by metformin (*p* value < 0.0001). Basal ECAR, indicative of glycolysis, on the other hand was slightly increased with metformin treatment (*p* value < 0.0001) (Figure 2B and 2D). Under induced energy demand in the presence of FCCP and oligomycin, OCR of stressed cells was higher compared to baseline OCR in both untreated and metformin-treated cells (≥1.5 fold increase for 0 μM, 100 μM, 1 mM of metformin, *p* value < 0.0001 for all, Figure 2A and 2C). However, under stress, metformin at a higher dose (1 mM) dampened the amplitude of increase in OCR levels as compared to ‘no treatment’ in Daudi cells only (*p* value < 0.0001 between stressed OCR at 0 μM vs. 1 mM; Figure 2A and 2C). The addition of stress compounds decreased ECAR levels, especially in Daudi cells with metformin exaggerating this effect slightly (Figure 2B and 2D). Taken together, our data show that the lymphoma cells generally prefer oxidative phosphorylation over glycolysis under stress and metformin treatment decreases oxygen consumption but does not completely reverse this preference. However, the observation that there is a dose-dependent increase in glycolytic flux due to metformin is notable and suggests that there might be an augmentation or modification of the Warburg effect in these cells.

**Figure 3:**
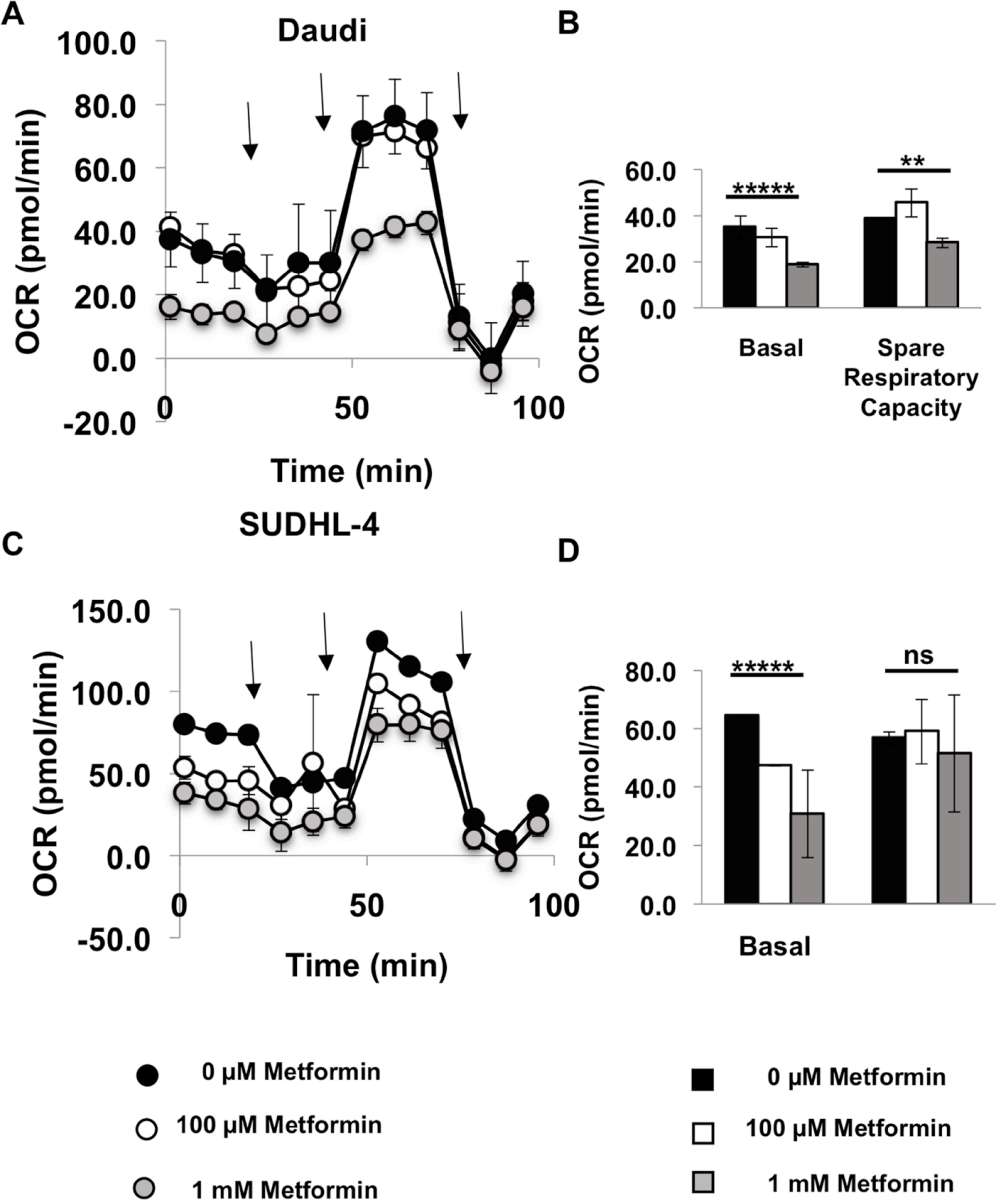
Metformin reduces oxygen consumption rate of lymphoma cells Daudi cells (**A**, **B**) and SUDHL-4 (**C**, **D**) were pretreated with 0 μM (Black), 100 μM (white), 1 mM (grey) metformin for 7 days and their metabolic state was determined using the Seahorse XF Cell Mito Stress test (Agilent). In short, after basal measurements, cells were stressed with 1 μM oligomycin (inhibits ATP synthase) first and OCR measurements were taken and then 0.5 μM FCCP for Daudi and 1 μM FCCP for SUDHL-4 cells (depolarizes mitochondrial inner membrane) was added and OCR measurements were taken and finally Antimycin A and rotenone were added together to inhibit mitochondrial respiration and OCR was measured again. No metformin was present during the assay (2 hours). Arrows in (A) and (C) represent addition of each stress compound. Basal respiration = (Last OCR measurement before oligomycin injection) – (nonmitochondrial respiration rate after antimycin A and rotenone injection). Spare respiratory capacity = (Maximal respiration after FCCP addition)-(Basal respiration). *p* values for basal OCR and spare respiratory capacity at 0 μM vs 1 mM metformin is shown. ^*****^p value < 0.0001; ^**^p value < 0.005; ns = not significant.

As lymphoma cells seem to prefer oxidative phosphorylation over glycolysis, we then performed the Seahorse XF Cell Mito Stress Test (Agilent) on metformin-pretreated Daudi and SUDHL-4 cells. After basal OCR measurements, cells were first stressed with oligomycin (inhibits ATP synthase), then FCCP (depolarizes mitochondrial inner membrane) and finally Antimycin A and rotenone (inhibit mitochondrial respiration). OCR was measured after each of these steps. As expected and observed from the Seahorse XF Cell Energy Phenotype Test kit, the Seahorse XF Cell Mito Stress Test also showed a significant reduction in basal OCR in both Daudi and SUDHL-4 cells when pretreated with 1 mM of metformin (*p* value < 0.0001 for both cell lines) and in addition spare respiratory capacity was reduced for the most sensitive cell line, Daudi (*p* value = 0.004; Figure 3). Altogether, these data demonstrate that there are significant metabolic changes occurring in cells treated with metformin at a concentration of 1 mM or higher.

### Treatment of lymphoma cells with metformin does not potentiate additive killing by conventional chemotherapy agent like doxorubicin

**Figure 4:**
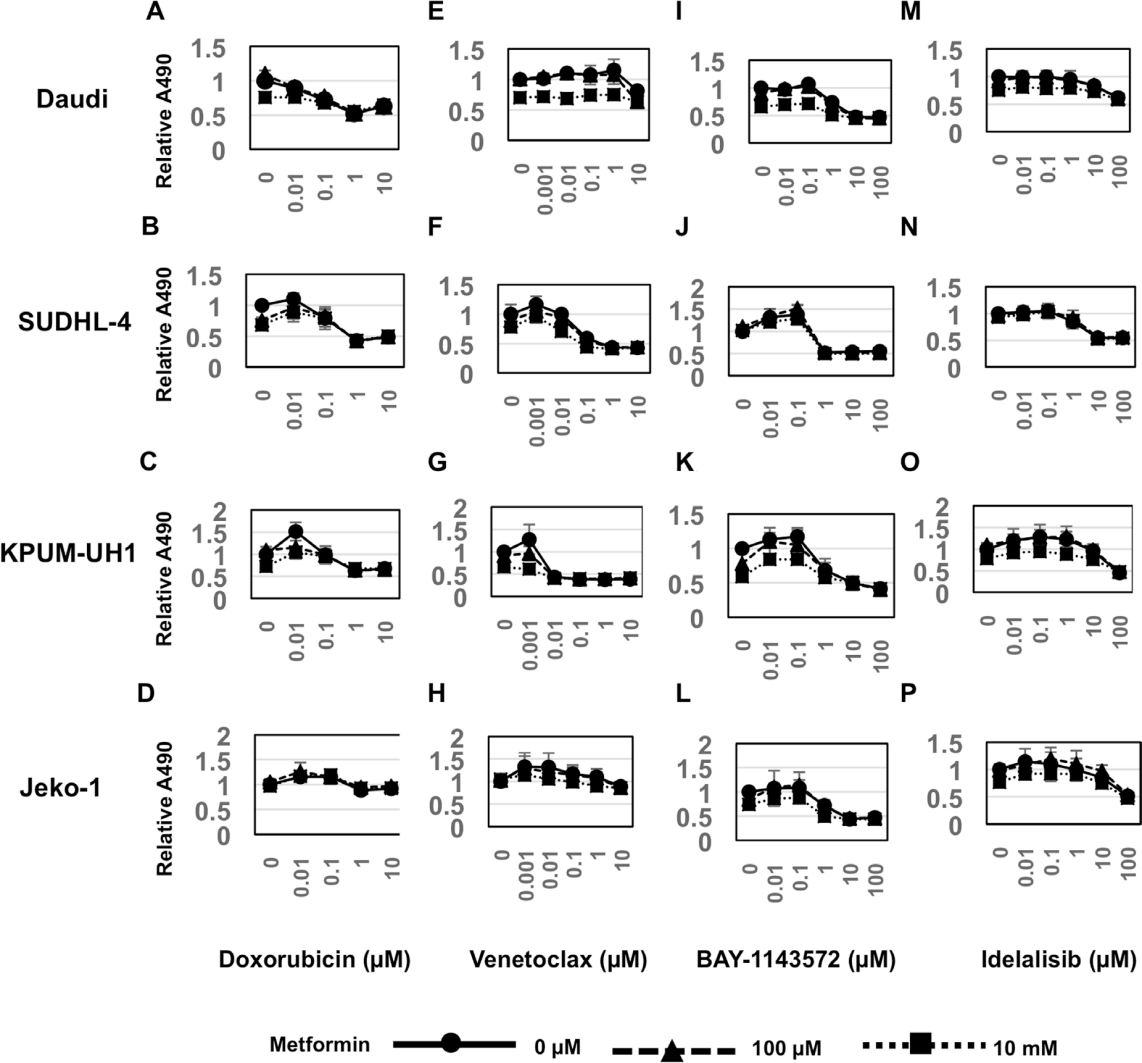
Metformin potentiates Venetoclax and BAY-1143572 in cell-type dependent manner 10,000 cells (**A**, **E**, **I**, Daudi, **B**, **F**, **J**. SUDHL-4, **C**, **G**, **K**. KPUM-UH1, **D**, **H**, **L**. Jeko-1) were plated per well of a 96-well plate and treated with dose response series of both metformin (results for concentrations 0, 100 μM,10 mM shown) and Doxorubicin (0–10 μM; Figure 4A–4D), or Venetoclax (0–10 μM; Figure 4E–4H) or BAY-1143572 (0–100 μM; Figure 4I–4L) or Idelalisib (0–100 μM; Figure 4M–4P) in triplicate. Viability after 3 days was analyzed using MTS assay. Briefly 20 μL of MTS reagent was added to cells and incubated for 2 hours and absorbance at 490 nm was read using a Biotek plate reader. Relative absorbance is calculated after setting the average absorbance of no treatment control as 1. Error bars represent standard deviation between triplicates.

To determine whether metabolic changes induced by metformin alter the viability of cells treated with conventional chemotherapy, we performed an MTS viability assay using dose-response series of both metformin and the anthracycline, doxorubicin. Ten thousand Daudi, SUDHL-4, KPUM-UH1 or Jeko-1 cells were plated in each well of a 96-well plate and treated in triplicate with a dose-response series of concentrations of both metformin (0 μM, 1 μM, 10 μM ,100 μM, 1 mM, 10 mM) and doxorubicin (0 μM, 0.01 μM, 0.1 μM, 1 μM, 10 μM). The number of cells at day 3 were quantified using MTS assay as indicated in the Methods section. As seen in Figure 4A and Supplementary Figure 1, although 10 mM of metformin treatment reduced viability of Daudi cells, it did not significantly alter the IC-50 value of Doxorubicin (Table1). Similarly, no potentiation of doxorubicin by metformin was observed in SUDHL-4, KPUM-UH1 and Jeko-1 cells (Figure 4B–4D, Supplementary Figure 2).

### Metformin shows potentiation with bcl-2 inhibitor Venetoclax and CDK9 inhibitor BAY-1143572 in cell-type dependent manner

To determine if metformin potentiates the anti-tumor activity of novel targeted agents, we tested the combination of metformin with either a bcl-2 inhibitor (Venetoclax) or CDK9 inhibitor (BAY-1143572) or PI3K inhibitor (Idelalisib). We treated Daudi, SUDHL-4, KPUM-UH1 or Jeko-1 cells simultaneously with a series of concentrations of metformin (0 μM, 1 μM, 10 μM, 100 μM, 1 mM, 10 mM) in combination with either Venetoclax (0 μM, 0.001 μM, 0.01 μM, 0.1 μM, 1 μM, 10 μM) or BAY-1143572 (0 μM, 0.01 μM, 0.1 μM, 1 μM, 10 μM, 100 μM) or Idelalisib (0 μM, 0.01 μM, 0.1 μM, 1 μM, 10 μM, 100 μM) and determined viability at day 3 using MTS assays. As seen in Figure 4E–4H and Supplementary Figure 2, SUDHL-4 and KPUM-UH1 cells were sensitive to Venetoclax, whereas Daudi and Jeko-1 cells were resistant at the concentrations tested. Combining Venetoclax with 10 mM of metformin had an apparent additive effect on cell killing in both the Venetoclax sensitive cell lines SUDHL-4 and KPUM-UH1 (Table 1). On the other hand, all cell lines tested were sensitive to BAY-1143572, (Figure 4I–4L and Supplementary Figure 3), but the benefit of adding metformin determined by its alteration of the IC-50 of BAY-1143572 was observed only in Daudi and Jeko-1 cells (Table 1). All cell lines other than Daudi were sensitive (reached IC-50) to Idelalisib at concentrations tested but no significant additive benefit was seen in the presence of metformin (Figure 4M–4P, and Supplementary Figure 4 and Table 1). Altogether, these data suggest that metabolic changes associated with metformin may potentiate Venetoclax and BAY-1143572 in a cell-type dependent manner. Please note that we explored several models including the Chou-Talay model to determine if metformin's effects were additive or synergistic in nature. These models did not provide a definitive categorization likely due to the large concentration ranges tested and an inability to reach IC-50 concentrations with metformin. We, therefore, use the term “potentiate” to indicate a benefit achieved with metformin; whether these effects are additive or synergistic remains indeterminate at this time.

**Table 1:** IC-50 values of drugs with and without 10 mM of metformin

	Daudi	SUDHL-4	KPUM-UH1	Jeko-1
**Doxorubicin alone**	~1 μM	~0.9 μM	NR	NR
**Doxorubicin with metformin**	~1 μM	~0.9 μM	NR	NR
**Venetoclax alone**	NR	**~0.8 μM**	**~0.01 μM**	ND
**Venetoclax with metformin**	NR	**~0.09 μM**	**~0.007 μM**	ND
**BAY 1143572 alone**	**~9 μM**	~1 μM	~10 μM	**~10 μM**
**BAY1143572 with metformin**	**~3 μM**	~1 μM	~10 μM	**~1 μM**
**Idelalisib alone**	NR	~10 μM	~100 μM	~100 μM
**Idelalisib with metformin**	NR	~10 μM	~100 μM	~100 μM

### Metformin increases caspase 3/7 activity in Venetoclax and BAY-1143572-treated lymphoma cells

**Figure 5:**
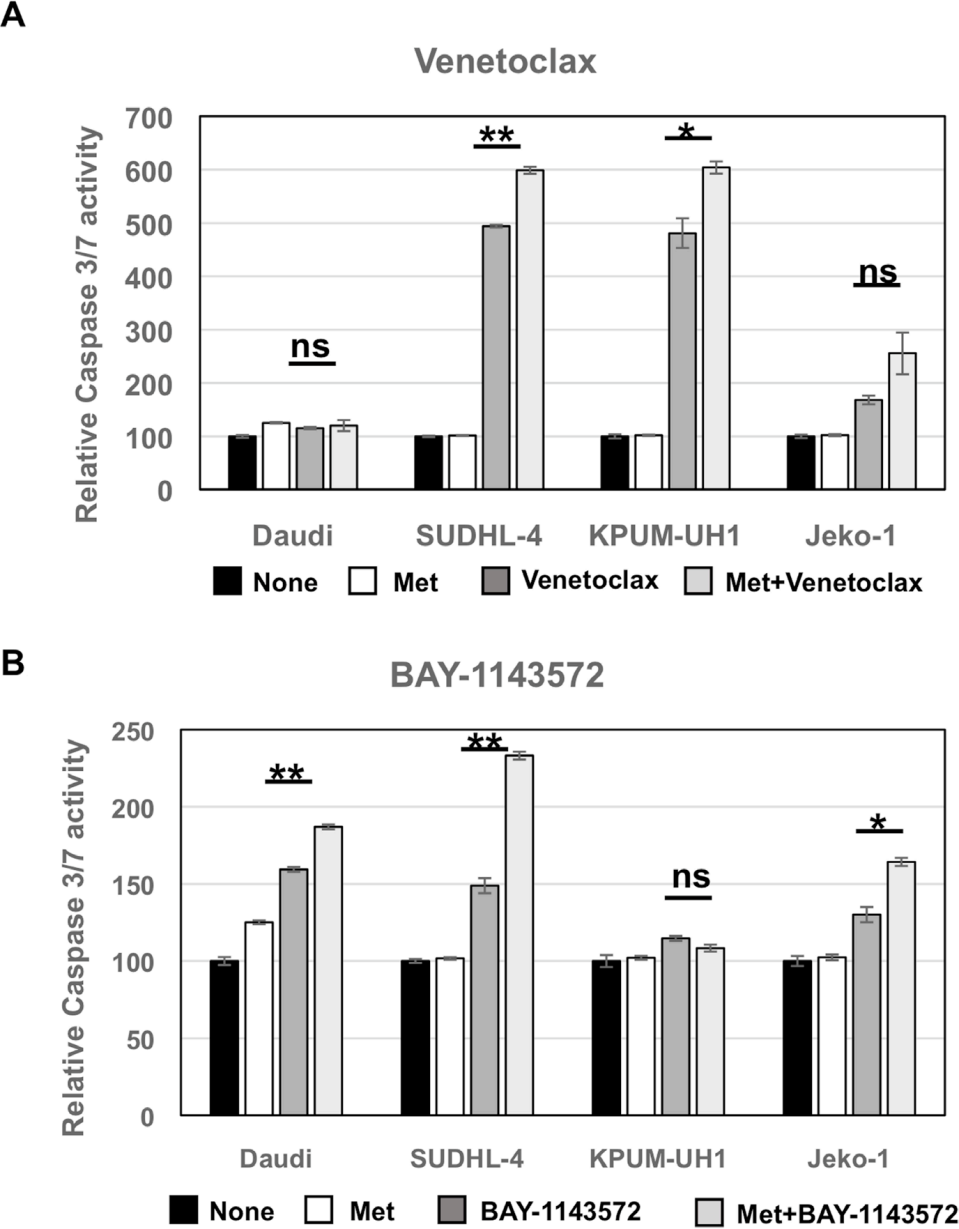
Metformin potentiates apoptosis-mediated cell killing by both Venetoclax and BAY-1143572 50,000 Daudi, SUDHL-4, KPUM-UH1 and Jeko-1 cells were left untreated or treated with 10,000 μM metformin or Venetoclax (0.01 μM for KPUM-UH1 and 0.04 μM for the other cells) or combination of metformin and Venetoclax for (**A**). or cells were left untreated or treated with 10 mM of metformin or 1 μM BAY-1143572 or the combination of both for (**B**). and after 4 hours were lysed and assayed for caspase 3/7 activity using EnzChek caspase 3 assay kit (ThermoFisher). The rhodamine 110-derived substrate (Z-DEVD-R110) used in this assay is a non-fluorescent bisamide compound that, upon enzymatic cleavage via active caspase 3 and may be caspase 7 in the cell lysates, is converted in a two-step process to the fluorescent monoamide and then to the even more fluorescent R110 product. Both of these products are then measured using Biotek synergy 2 fluorescent plate reader at corresponding wavelengths (excitation 496 nm/emission 520 nm). The fluorescence reading of untreated control for each cell line was set to 100% and was used to normalize the rest of the data. *p* values comparing Venetoclax/BAY-1143572 with or without 10 mM metformin is shown. ^**^p < 0.005; ^*^p < 0.05; ns = not significant.

To determine the cell-death pathways promoted by metformin, Daudi, SUDHL-4, KPUM-UH1 and Jeko-1 cells were left untreated or treated with 10 mM of metformin or Venetoclax (0.01 μM for KPUM-UH1 and 0.04 μM for the rest) or a combination of metformin and Venetoclax. After 4 hours, cells were lysed and assayed for caspase 3/7 activity as detailed in Materials and Methods. As seen in Figure 5A, metformin alone did not change caspase 3/7 activity significantly while Venetoclax monotherapy significantly enhanced caspase 3/7 activity in SUDHL-4 and KPUM-UH1 cells (*p* value < 0.0001 and *p* value 0.0027, respectively). There was a slight increase in caspase 3/7 activity with Venetoclax treatment in Jeko-1 (low bcl-2 expressing) cells and no increase in Daudi (bcl-2 non-expressing) cells, both of which are resistant to Venetoclax at the concentration tested. This caspase 3/7 activity mediated by Venetoclax was further enhanced in the presence of 10 mM metformin (*p* value = 0.002 for SUDHL-4 and 0.029 for KPUM-UH1 comparing Venetoclax alone to Venetoclax with metformin) supporting the potentiation observed previously and shown in Figure 4. Similarly, cells were left untreated or treated with 10 mM metformin or 1 μM BAY-1143572 or the combination of both. As seen in Figure 5B, all cells except KPUM-UH1 showed an increase in caspase 3/7 activity in BAY-1143572 treated cells. This caspase3/7 activity was further enhanced when cells were treated with metformin + BAY-1143572 (*p* value = 0.003 for Daudi, 0.002 for SUDHL-4 and 0.01 for Jeko-1 comparing BAY-1143572 alone to BAY-1143572 with metformin). Altogether, these data show that metformin potentiates apoptosis-mediated cell killing by both Venetoclax and BAY-1143572.

### Metformin potentiation of Venetoclax and BAY-1143572 is AMPK-independent

**Figure 6:**
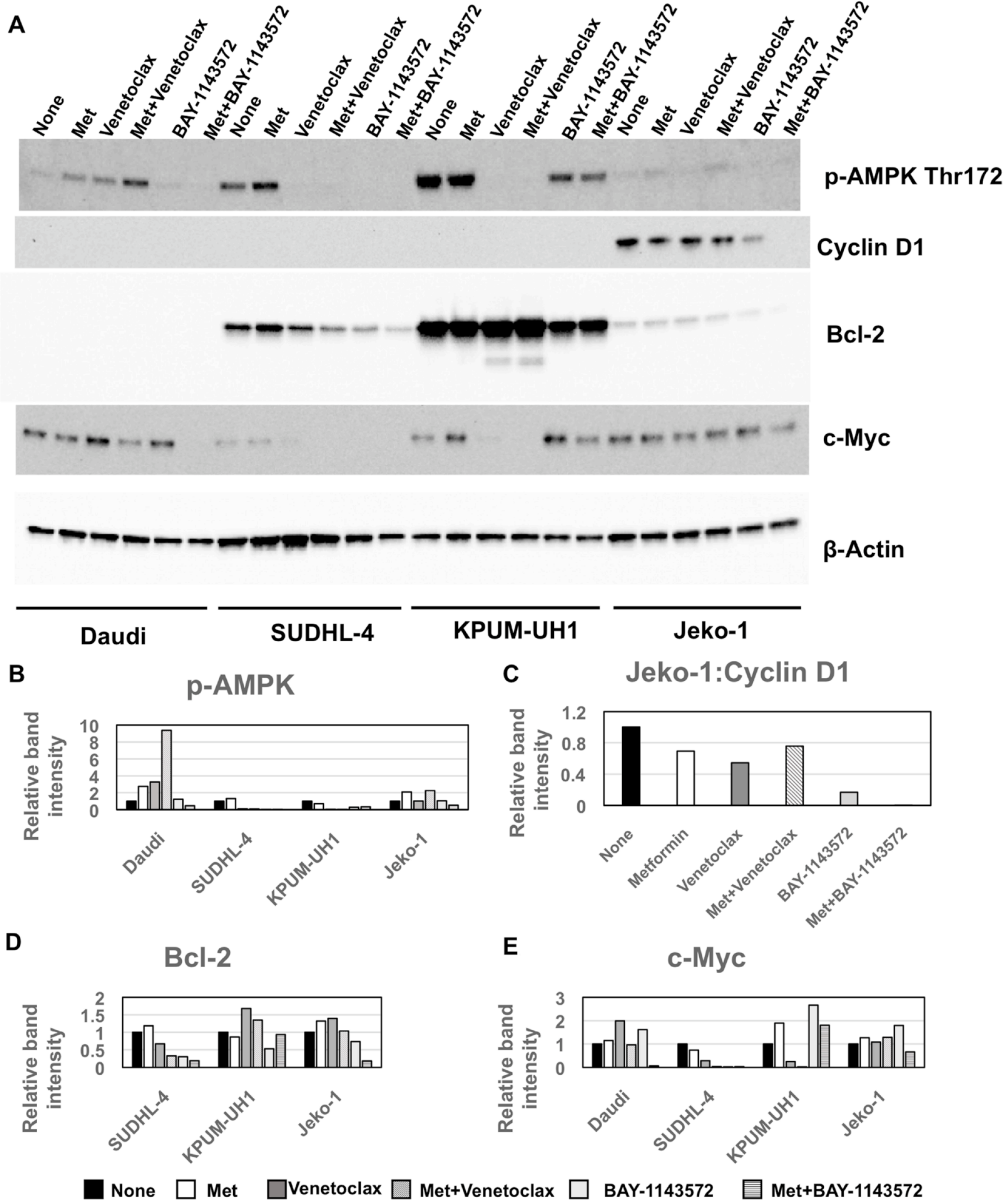
Metformin mediated potentiation of Venetoclax and BAY-1143572 is AMPK independent (**A**) 10 million cells were left untreated or treated with 10 mM of metformin or Venetoclax (0.01 μM for KPUM-UH1 and 0.04 μM for the rest) or combination of metformin and Venetoclax or 1 μM of BAY-1143572 or the combination of both for 48 hours and were then spun down at 200 rcf for 5 minutes and rinsed once with PBS before lysing in 50 μL of Millipore Milliplex MAP lysis buffer supplemented with protease and phosphatase inhibitors (Roche). Around 20–30 μg of protein was loaded per well and analyzed via Western blotting for p-AMPK-alpha (Thr 172), Cyclin D1, bcl-2, c-MYC and β- actin (control) as stated in methods section. Bands were quantified and normalized to total protein levels obtained via stain-free gel image and graphed in (**B**) for p-AMPK-alpha (Thr 172), (**C**) for Cyclin D1, (**D**) for bcl-2, (**E**) for c-MYC.

To determine the mechanism by which metformin potentiates Venetoclax and BAY-1143572 effects, we treated Daudi, SUDHL-4, KPUM-UH1 and Jeko-1 cells with single agent metformin, or Venetoclax (0.01 μM for KPUM-UH1 and 0.04 μM for the rest) ± 10 mM metformin or 1 μM of BAY-1143572 ± 10 mM metformin for 48 hours. Changes in signaling molecules as compared to untreated control were analyzed via western blotting as described in Materials and Methods. The molecules analyzed include p-AMPK (Thr172), bcl-2, cyclin D1, c-MYC and the graphs were normalized to total protein analyzed via stain-free imaging technology (Bio-Rad). As seen in Figure 6, treating cells with metformin increased p-AMPK (Thr172) levels in all cell lines except KPUM-UH1 cells. By contrast, treating cells with Venetoclax or BAY-1143572 significantly reduced p-AMPK (Thr172) levels in sensitive cell-lines with potentiation of this effect upon addition of metformin. Of note, in Venetoclax resistant cells, p-AMPK (Thr172) levels were either increased (Daudi) or unchanged (Jeko-1) and the addition of metformin further enhanced these levels. Thus, metformin-mediated potentiation of cell killing in Venetoclax and BAY-1143572 treated cells seems to be independent of activation of p-AMPK. In the bcl-2 expressing cells (SUDHL-4, KPUM-UH1 and Jeko-1), metformin treatment alone did not change bcl-2 levels significantly. However, the alterations in bcl-2 protein were cell-type and treatment-dependent for all other conditions. Specifically, in SUDHL-4 cells, Venetoclax treatment and BAY-1143572 treatment reduced bcl-2 levels and this effect was enhanced with the addition of metformin. In Venetoclax resistant Jeko-1 cells, treatment with BAY-114372 but not Venetoclax altered bcl-2 levels and metformin potentiated this reduction. In KPUM-UH1 cells, BAY-1143572 reduced bcl-2 levels with no added effect upon metformin exposure. Although Venetoclax treatment in KPUM-UH1 cells did not alter bcl-2 levels, closer observation revealed both higher molecular weight laddering and a low molecular weight band suggestive of some post-translational modification, ubiquitination as a possibility (Supplementary Figure 5). This process was significantly enhanced in the presence of metformin.

Cyclin D1 was highly expressed only in Jeko-1. BAY-1143572 treatment reduced cyclin D1 expression significantly as to be expected and metformin exposure further potentiated this reduction; Venetoclax had no effect. C-MYC protein levels were also altered in a cell-type dependent and drug-dependent manner. Metformin alone did not change c-MYC expression in cells while Venetoclax reduced c-MYC levels significantly in sensitive cell lines, SUDHL-4 and KPUM-UH1. Though not significant, metformin did potentiate the reduction of c-MYC in these Venetoclax treated cells. BAY-114372 treatment reduced c-MYC levels only in SUDHL-4 cells (IC-50 of 1 μM), but not in other cell lines tested (IC-50: ~10 μM), not surprising given the low concentration of the agent used (1 μM). However, the combination of metformin and BAY-1143572 significantly reduced c-MYC expression compared to untreated cells in both Daudi and Jeko-1, in keeping with viability data obtained for this combination. Altogether, these data confirm that metformin potentiates the signaling pathways targeted by both Venetoclax and BAY-1143572 in a cell-type dependent manner, independent of metformin's ability to activate AMPK.

## DISCUSSION

We investigated the effects of single agent metformin on cellular metabolism and viability in aggressive B-cell lymphoma cell lines of varying histology and biology. We also explored the effect of metformin used in combination with chemotherapy and novel targeted agents, Venetoclax, BAY-1143572 and Idelalisib, each chosen for their unique tumorigenic effects and concomitant influences on cellular metabolism.

We found that metformin as a single agent had differential effects on lymphoma cell lines. Although metformin did not impact viability or proliferation in KPUM-UH1 and Jeko-1 cells, the drug did alter viability in Daudi and SUDHL-4 cell lines with effects on basal oxygen consumption rates, suggesting a therapeutic role for targeting cellular metabolism in specific lymphoma subsets. We did not conduct studies in non-germinal center cell lines to determine differential effect according to cell of origin.

The effect of metformin on cell lines tested was only observed with 1 mM or higher concentrations of metformin. Additionally, metformin did not potentiate the effects of the anthracycline doxorubicin as previously seen by Shi WY *et al.* [19]. It should be noted that 10 mM and higher concentrations of metformin were used in their study and doxorubicin concentration tested was unclear. In our study, a maximum of 10 mM Metformin and 10 μM doxorubicin were used and could be a reason for no potentiating effect seen. Additionally, in the Shi paper [19], the effects of these agents observed *in vivo* with xenograft models may be attributed to chronic exposure of metformin given orally daily for 14 days with doxorubicin dosed twice weekly. These findings raise the question of whether chronic exposure to metformin could circumvent the need for higher concentrations of this agent or whether a more potent biguanide such as phenformin should be evaluated for anti-tumor effect in combination with chemotherapy as a chemotherapy sparing approach [27]. The latter however, may carry greater risks of adverse effects such as lactic acidosis [28].

The bcl-2 inhibitor, Venetoclax, as a single agent in non-Hodgkin Lymphoma has shown quite variable activity. In a Phase I trial of a 106 patients (MCL; *n* = 28, FL; *n* = 29 , DLBCL; *n* = 34), overall response rate was only 44% (MCL, 75%; FL, 38%; DLBCL, 18%) with an estimated median progression free survival of 6 months, requiring doses as high as 800 mg in MCL and 1200 mg in the DLBCL for efficacy [29]. Strategies that could potentiate the anti-tumor effects of Venetoclax are of interest serving as the rationale for our metformin combination studies.

We showed that metformin, notably at a high concentration, when combined with Venetoclax, increased sensitivity of KPUM-UH1, a double hit cell line that is typically chemotherapy resistant, and SUDHL-4, a germinal center origin DLBCL cell line, to the bcl-2 inhibitor. Specifically, viability was decreased while caspase 3/7 activity was increased in cells treated with this combination compared to Venetoclax alone. Our western blot data suggest that the potentiating effects of metformin to Venetoclax are however independent of its activity on AMPK and perhaps even c-MYC in c-MYC positive cells.

For the first time, we also demonstrated that high concentration metformin increased sensitivity of lymphoma cells to the CDK9 inhibitor, BAY-1143572 with potentiating effects in the mantle cell line Jeko-1 of particular interest. Cyclin-dependent kinases are universally overactive in lymphoma and represent potential anti-neoplastic drug targets. Whether pan-CDK inhibition is superior for inducing anti-tumor effects as compared to selective CDK inhibition has been widely debated. Metformin has been shown to block cyclin D1, Cdk4 and Cdk6 in various cancers [30–32]. Our western blot data similarly suggest that metformin's augmentation of BAY-1143572 activity in the MCL cell line are related to the broader CDK inhibition achieved with the combination rather than metformin's ability to activate AMPK.

Interestingly, metformin did not have any potentiating effect when combined with PI3K inhibitor, Idelalisib, likely representing redundancy of effect on the PI3K/Akt pathway in lymphoma [33, 34]. Collectively, although metformin's effects on AMPK have been well described [35, 36], our data challenges the relevance of this pathway in specific lymphoma histologies but is in keeping with results reported by others suggesting that AMPK is not required for the anti-proliferative effects of metformin [37].

Despite our attempts, the optimal dose of metformin to elicit anti-lymphoma effects remains unclear and seemingly physiologically unachievable. We were only able to show anti-tumor effects of monotherapy metformin at concentrations of 1 mM or higher. Similarly, the potentiating effects of metformin to novel agents were demonstrated at a concentration of 10 mM. For mechanistic studies (western blots and caspase assays), we did not attempt combination experiments with lower doses of metformin given millimolar range concentrations reported by other investigators in cell lines [10] and *in vitro* models [38, 39]. However, pharmacokinetic studies of metformin in diabetic patients suggest achievable levels of less than 20 μM with doses of up to 3000 mg of metformin [40]. As changes in viability and proliferation are dependent on concentrations of at least 1 mM of metformin in our studies, it is unclear that physiologically achievable doses of metformin could have anti-tumor effects. This is in contrast to significant body of epidemiologic literature suggesting an anti-cancer therapeutic benefit to metformin exposure. These observations lend support to the notion that perhaps, chronic exposure to the agent might circumvent the need for higher serum concentrations for anti-tumor effect [21, 22, 41]. Additionally, higher concentrations used for *in vitro* experiments may be attributed to the use of glucose-rich media—there is some suggestion that such concentrations may not be needed for similar effects *in vivo* [42–44]. Nonetheless, careful consideration for metformin concentration and chronicity of exposure is required for ongoing clinical trials looking at short courses of metformin as a single agent or in combination with cytotoxics or radiation.

**Figure 7:**
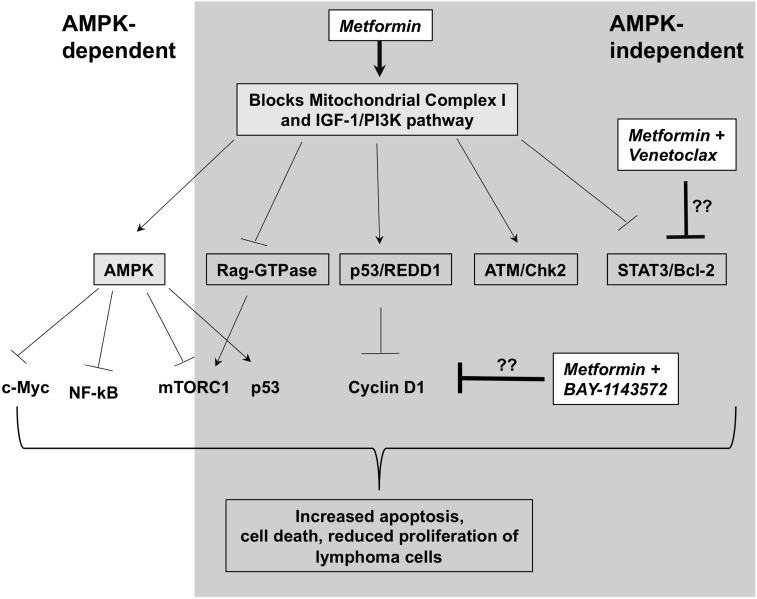
Possible mechanism of action of metformin in lymphoma cells Metformin blocks mitochondrial complex I leading to activation of AMPK via reduction in ATP production. This in turn, leads to activation and inactivation of several pathways dependent on AMPK. Only some of the relevant molecules are shown. In addition, metformin blocks or activates several pathways independent of AMPK. Some of them include inactivating PI3K/AKT pathway, activating DNA damage response (ATM/Chk2 and p53) pathways, inhibiting Rag-GTPases that activate mTOR. Venetoclax, a bcl-2 inhibitor is potentiated by metformin likely via it's role in blocking Stat3 pathway independent of AMPK and BAY-1143572 is likely potentiated by metformin via it's role in reducing cyclin D1 independent of AMPK. Though, involvement of other pathways in the potentiation of these drugs can't be ignored. All these signaling changes with metformin treatment eventually lead to increase in apoptosis-mediated cell death and reduction in cell proliferation of lymphoma cells. However, it should be noted that depending on the lymphoma cell type, some of these pathways may be more active and some pathways may be missing or dysregulated altogether.

In summary, our study represents the first to explore the concept of targeting cancer metabolism with metformin in novel combinatorial therapeutic strategies for lymphoma. We show that metformin induces significant changes in cellular metabolism, a mechanism that may lead to decreased proliferation and viability in some lymphoma cell lines (Summarized in Figure 7). Our metformin-Venetoclax and metformin-BAY-1143572 combination studies uncover metformin's broader and differential anti-lymphoma activity, not initially identified in experiments with single agent metformin studies. We show that metformin exerts anti-tumor activity through variable mechanisms of action, some of which may be independent of its effects on cellular metabolism and the AMPK/mTOR pathway. However, these anti-lymphoma effects of metformin are reliant on the presence of higher concentrations of the agent *in vitro* which may be physiologically prohibitory *in vivo*. Ongoing metformin based prospective studies may clarify this concern.

## MATERIALS AND METHODS

### Routine culture of cell lines

Daudi (Burkitt; Cat. No: CCL-213, Lot. No: 60754101), SUDHL-4 (germinal center DLBCL; Cat. No: CRL-2957, Lot.No: 62612711) and Jeko-1 (Mantle cell lymphoma; Cat. No: CRL-3006, Lot. No: 62280070) cells were purchased from American Type Culture Collection (ATCC) whereas KPUM-UH1 (double hit DLBCL cells) cells were a kind gift from Junya Kuroda, Division of Hematology and Oncology, Department of Medicine, Kyoto Prefectural University of Medicine, Kyoto, Japan [45]. All cells were maintained in RPMI-1640 (corning) containing 0.3 g/mL glutamine, 10% FBS (Sigma), and antibiotic and anti-mycotic reagent (Gemini Bioproducts; final concentrations, 100 units/mL penicillin G, 100 μg/mL streptomycin sulfate, and 250 ng/mL amphotericin B).

### Drugs

Metformin was purchased from Calbiochem and re-suspended in cell-culture media as required. Doxorubicin, Venetoclax and Idelalisib were purchased from Selleck Chemicals and re-suspended in DMSO. BAY-1143572 was purchased from Active Biochem.

### Hoechst 33342 assay

After plating appropriate cells in a U-bottom 96 well plate and treating them as indicated in the Results section, cells were spun at 100 rcf for 3 minutes to remove most of the supernatant, washed 1× with PBS, spun down again to remove the supernatant and 50 μL PBS was added per well. Cells were then freeze-thawed 3 times to lyse and 50 μL of 5 μg/mL Hoechst 33342 (Thermo Fisher Scientific) was added and incubated at RT for 30 minutes. Fluorescence reading was taken using Biotek Synergy 2 fluorescent plate reader at corresponding wavelengths (excitation 350 nm/emission 461 nm). Fluorescence reading of Hoechst 33342 at 0 μM of metformin on all days was set to 100% and was used to normalize the rest of the data.

### Seahorse metabolic assays (Agilent technologies)

Cell numbers were optimized for each cell line individually using Glycolysis Stress Test Kit per manufacturer instructions. For Daudi, 2.5 × 10^5^ cells per well were used and for SUDHL-4, 3 × 10^5^ cells were used unless otherwise stated. FCCP was titrated for each cell line using Mito stress test kit as instructed by manufacturer −0.5 μM of FCCP for Daudi and 1 μM of FCCP for SUDHL-4 was considered appropriate. Using the above parameters, cells pretreated with 100 μM and 1 mM of metformin for 7 days were analyzed using the Seahorse XF Cell Energy Phenotype Kit as instructed by manufacturer. Briefly Seahorse XF24 cell culture plates were coated with 50 μL per well of neutralized Cell-Tak solution (Corning; 22.4 μg/mL), incubated for 20 minutes and washed with water and air dried. Corresponding amount of pre-treated lymphocyte cells were plated in 100 μL culture medium and centrifuged at 200 rcf for 1 minute with zero braking for adhering the cells to the plate. Cells were then incubated at 37° C in a non-CO_2_ incubator for 25–30 minutes and 400 μL of warm medium was further added gently to the wells and returned back to the non-CO_2_ incubator for additional 15–25 minutes. The plate was then placed in the seahorse XF24 analyzer when prompted. XF cartridge was hydrated overnight and the port A was filled with the mixture of trifluoromethoxy carbonylcyanide phenylhydrazone (FCCP) and oligomycin (1 μM final concentration) to induce stress and calibrated before analyzing the cells. Cell-energy phenotype test kit measures both baseline oxygen consumption rate (OCR) and extracellular acidification rate (ECAR) and measurements after combination of FCCP and oligomycin stress and the results were analyzed using Cell-energy phenotype report generator.

Similarly, cells plated as above were also analyzed using the Seahorse XF Mito Stress Test kit where the cartridge port A was filled with oligomycin (final conc. 1 μM), port B with FCCP (final concentration of 0.5 or 1 μM) and port C with antimycin A/rotenone (final concentration of 0.5 μM). After basal OCR measurements, OCR was then measured after sequential injection of drugs from ports A, B and C to determine the changes associated with the corresponding reagent. The data was then analyzed using the associated report generators. Oligomycin inhibits ATP synthase and thus brings down OCR that is associated with ATP production, FCCP depolarizes mitochondrial membrane and uncouples OCR from ATP production and increases OCR to maximal level while antimycin A/rotenone inhibit electron transport chain complexes I and III and thus inhibit mitochondrial OCR completely.

### MTS assay

(3-(4,5-dimethylthiazol-2-yl)-5-(3-carboxymethoxyphenyl)-2-(4-sulfophenyl)-2H-tetrazolium) (MTS) assay was performed using Promega CellTiter 96^®^ AQ_ueous_ One Solution Cell Proliferation Assay reagent as per manufacturer instructions. Briefly, at the end of the treatment, 20 μL of reagent was added per well in 96-well plate and incubated for 2–4 h at 37° C. The absorbance at 490nm was determined using Biotek plate reader.

### EnzChek^®^ caspase 3 assay (Thermofisher scientific)

Caspase assay was performed as per manufacturer's instructions. Briefly, 50000 cells were plated in a 24-well plate and treated with indicated drugs for 4 hours in duplicate. At the end of treatment, cells were centrifuged at 200 rcf for 5 minutes and washed once with 1× PBS and lysed in 50 μL of 1× lysis buffer provided in the kit. For efficient lysis, cells were subjected to a single freeze-thaw cycle. The lysed cells were centrifuged again to remove cell debris and the supernatant was used in the assay. 50 μL of 2× substrate working solution containing Z-DEVD-R110 substrate was added to the cell lysate and incubated at room temperature for 30 minutes. The rhodamine 110-derived substrate (Z-DEVD-R110) used in this assay is a non-fluorescent bisamide compound that, upon enzymatic cleavage via active caspase 3 and may be caspase 7 in the cell lysates, is converted in a two-step process to the fluorescent monoamide and then to the even more fluorescent R110 product. Both of these products are then measured using Biotek synergy 2 fluorescent plate reader at corresponding wavelengths (excitation 496 nm/emission 520 nm). The fluorescent reading was then normalized to the amount of protein in the cell lysate determined via standard BCA assay (Pierce ThermoFisher Scientific).

### Western blotting

10 million cells were treated with various conditions for 48 hours and were then spun down at 200 rcf for 5 minutes and rinsed once with PBS before lysing in 50 μl of Millipore Milliplex MAP lysis buffer supplemented with protease and phosphatase inhibitors (Roche). After BCA analysis for determining protein concentration, 15–40 μg of protein denatured in 4× sample buffer supplemented with β-mercaptoethanol (Bio-Rad) was loaded per well depending on the antibody tested. Bio-Rad stain-free Criterion 4–20% precast gels were used. After running the gel at 140 volts for 90 minutes, Bio-Rad gel imager was used to activate the stain-free technology to visualize the total protein levels loaded in the gel. Nitrocellulose turbo-transfer pack and system (Bio-Rad) was then used to transfer the proteins to the membrane and 5% w/v dry milk in Tris-buffered saline-0.1% Tween 20 (TBS-T) was used to block the membranes for 1 hour. Membranes were then incubated with primary antibody diluted in 5% BSA in TBS-T overnight. Membranes were then rinsed in TBS-T 3 times (one 15 minute wash and two 5 minutes washes) and then incubated with corresponding secondary antibody-conjugated with HRP for 1 hour and rinsed with TBS-T as before. After final wash, membranes were developed using Pierce Super signal West Pico chemiluminescence kit and visualized using Bio-Rad imaging system. In addition to chemiluminescent image, the stain free-image of total protein was also taken to normalize the protein bands. When necessary, membranes were stripped with Pierce stripping solution and re-blocked and re-probed.

Included: Rabbit p-AMPK alpha (Thr 172; Cat. No: 2535), Rabbit c-Myc (Cat. No: 5605), Rabbit Cyclin D1 (Cat. No: 2978), Mouse bcl-2 (Cat. No: 15071) and were purchased from Cell Signaling and used at 1:1000 dilution. Mouse Beta-Actin (Santa Cruz, Cat. No: sc-47778; 1:500) was used as a loading control. Anti-Rabbit HRP and Anti-Mouse HRP secondary antibodies were purchased from Cell Signaling and used at 1:5000 dilution.

### Statistical analysis

Student's *t*-test was performed to determine significance. Error bars represent standard deviation. *p*-values of less than 0.05 were deemed statistically significant.

## SUPPLEMENTARY MATERIALS FIGURES



## Abbreviations

AMPK: Adenosine monophosphate-activated protein kinase
DLBCL: diffuse large B-cell lymphoma
CDK: cyclin-dependent kinase
DHL: double-hit lymphoma
MCL: Mantle cell lymphoma
