# Supplementary material for: Metabolic changes associated with metformin potentiates Bcl-2 inhibitor, Venetoclax, and CDK9 inhibitor, BAY1143572 and reduces viability of lymphoma cells
Source: Oncotarget. 2018 Apr 20;9(30):21166–81. doi: 10.18632/oncotarget.24989 (PMC5940409; doi:10.18632/oncotarget.24989)
Supplement: Supplementary file 1 [file oncotarget-09-21166-s001.pdf]

Metabolic changes associated with metformin potentiates Bcl-2 inhibitor, Venetoclax, and CDK9 inhibitor, BAY1143572 and reduces viability of lymphoma cells

SUPPLEMENTARY MATERIALS

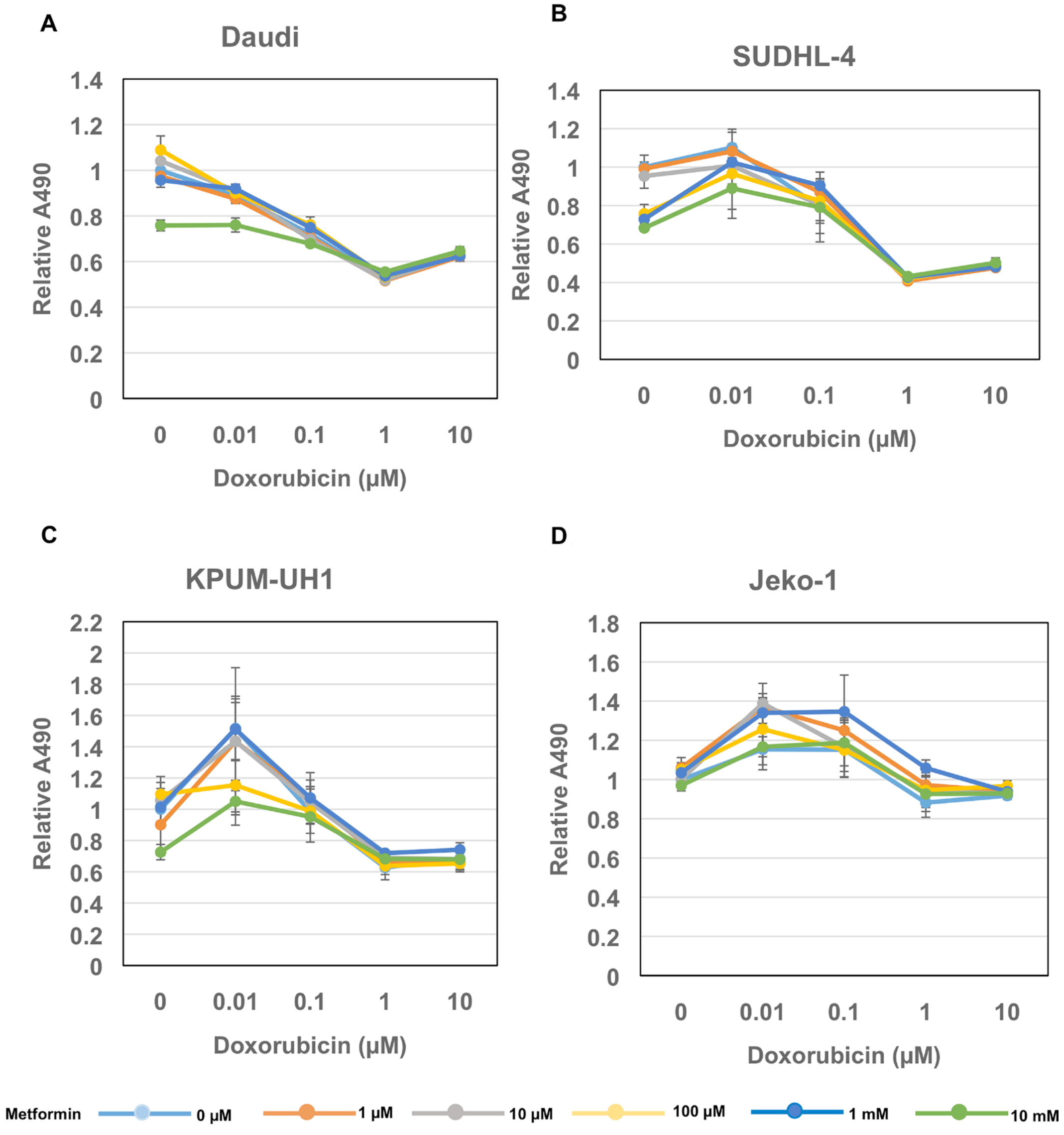

Supplementary Figure 1: Metformin does not potentiate Doxorubicin. 10,000 cells (**A** Daudi, **B** SUDHL-4, **C** KPUM-UH1, **D** Jeko-1) were plated per well of a 96-well plate and treated with dose response series of both metformin (0–10 mM) and Doxorubicin (0–10 µM). Viability after 3 days was analyzed using MTS assay. Briefly 20 µL of MTS reagent was added to cells and incubated for 2 hours and absorbance at 490 nm was read using biotek plate reader. Relative absorbance is calculated after setting the average absorbance of No tx ctrl as 1.

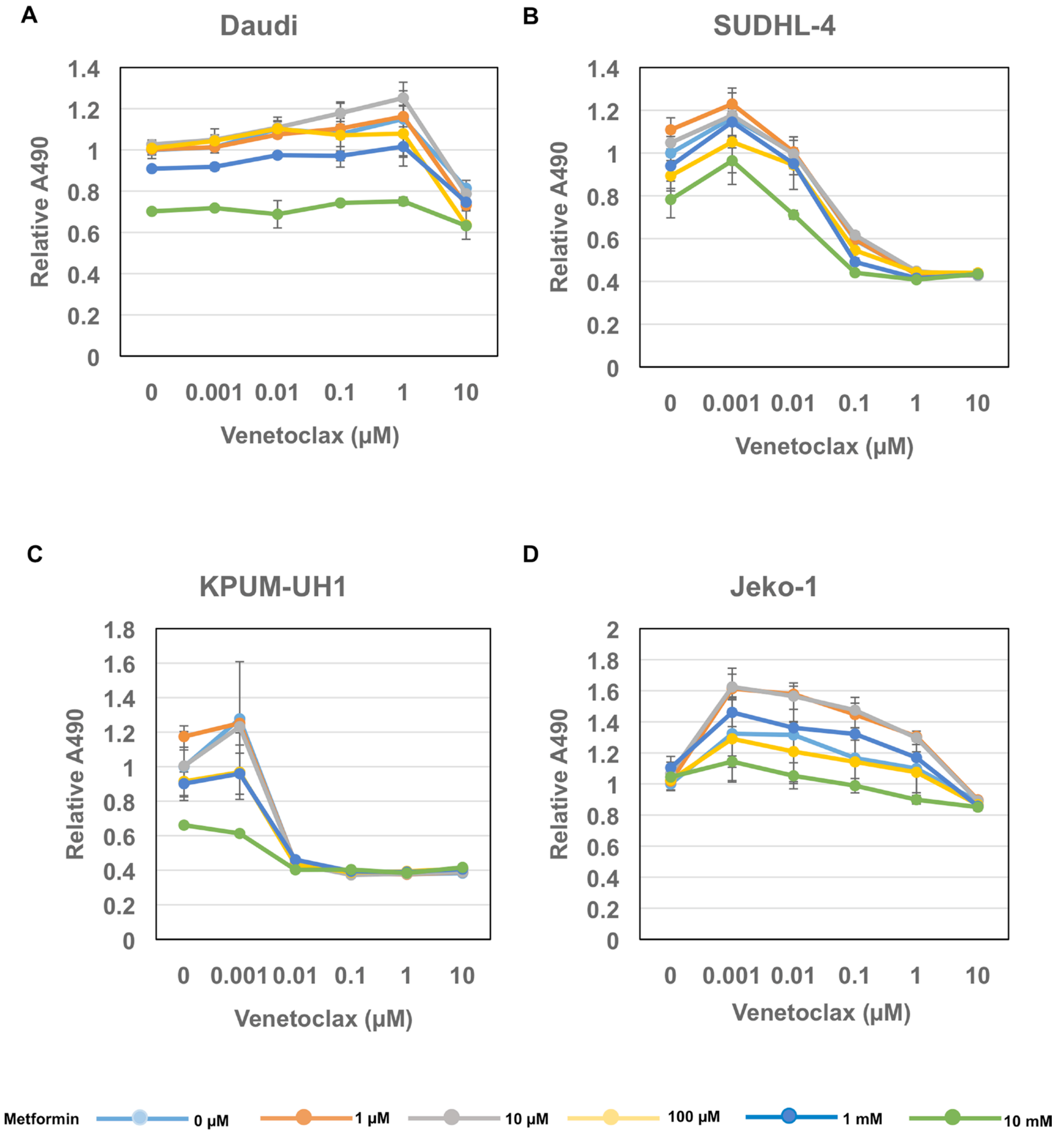

Supplementary Figure 2: Metformin potentiates Venetoclax in cell-type dependent manner. 10,000 cells (**A** Daudi, **B** SUDHL-4, **C** KPUM-UH1, **D** Jeko-1) were plated per well of a 96-well plate and treated with dose response series of both metformin (0–10 mM) and Venetoclax (0–10 nM) Viability after 3 days was analyzed using MTS assay. Briefly 20 µL of MTS reagent was added to cells and incubated for 2 hours and absorbance at 490 nm was read using biotek plate reader. Relative absorbance is calculated after setting the average absorbance of No tx ctrl as 1.

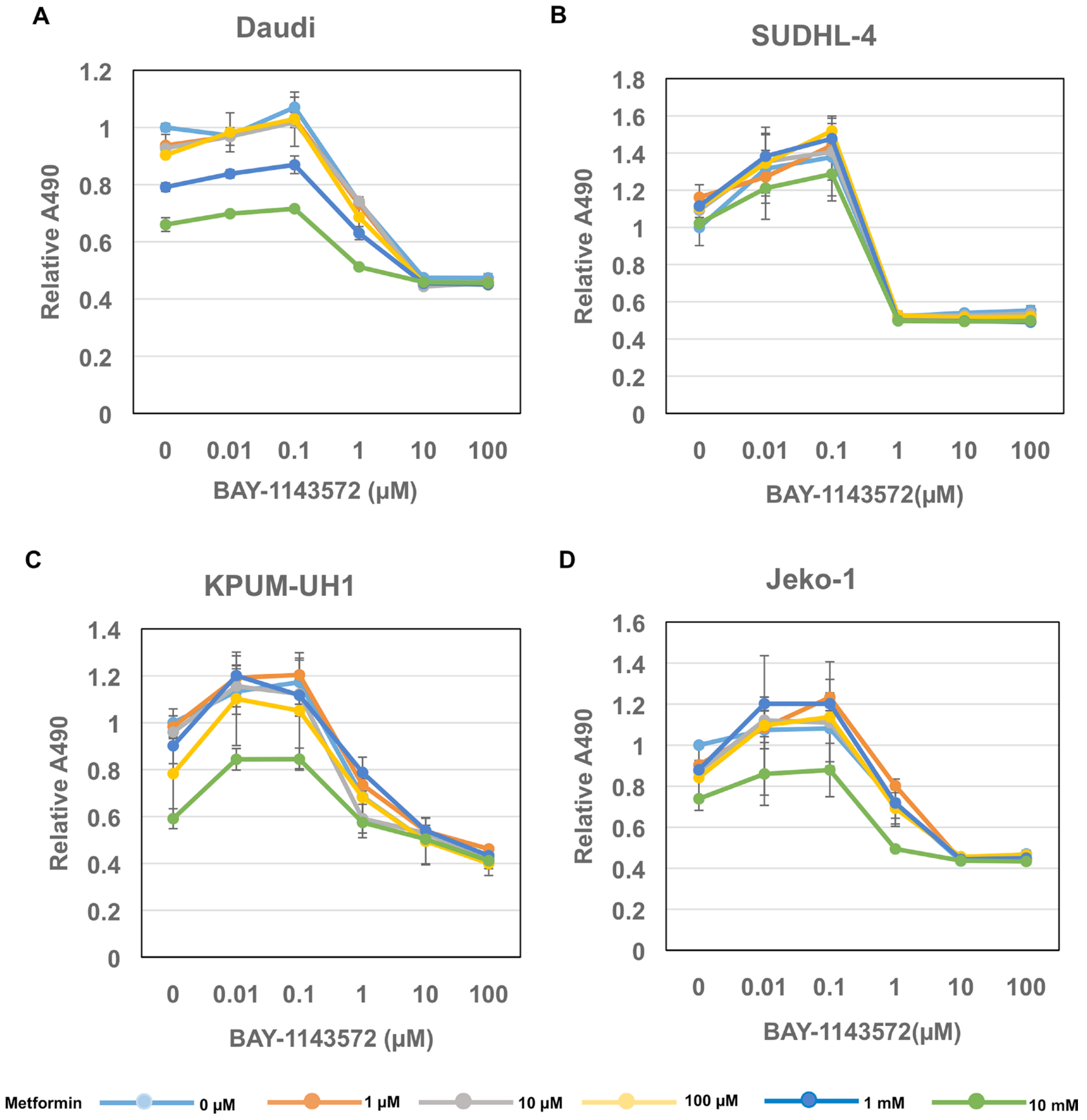

Supplementary Figure 3: Metformin potentiates BAY-1143572 in cell-type dependent manner. 10,000 cells (**A** Daudi, **B** SUDHL-4, **C** KPUM-UH1, **D** Jeko-1) were plated per well of a 96-well plate and treated with dose response series of both metformin (0–10 mM) and BAY-1143572 (0–100 µM). Viability after 3 days was analyzed using MTS assay. Briefly 20 µL of MTS reagent was added to cells and incubated for 2 hours and absorbance at 490 nm was read using biotek plate reader. Relative absorbance is calculated after setting the average absorbance of No tx ctrl as 1.

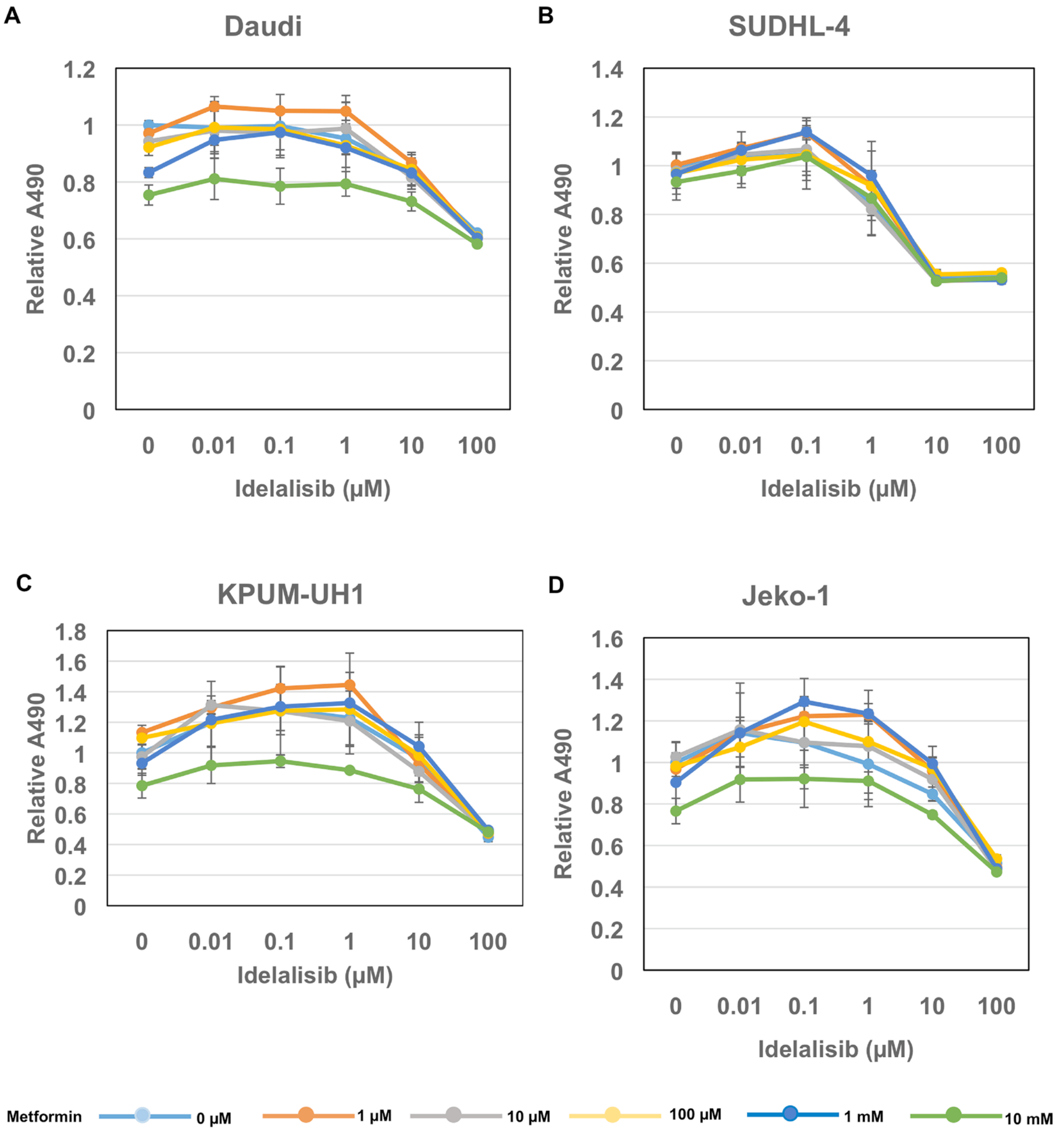

Supplementary Figur 4: Metformin does not potentiate Idelalisib. 10,000 cells (**A** Daudi, **B** SUDHL-4, **C** KPUM-UH1, **D** Jeko-1) were plated per well of a 96-well plate and treated with dose response series of both metformin (0–10 mM) and Idelalisib (0–100 µM). Viability after 3 days was analyzed using MTS assay. Briefly 20 µL of MTS reagent was added to cells and incubated for 2 hours and absorbance at 490nm was read using biotek plate reader. Relative absorbance is calculated after setting the average absorbance of No tx ctrl as 1.

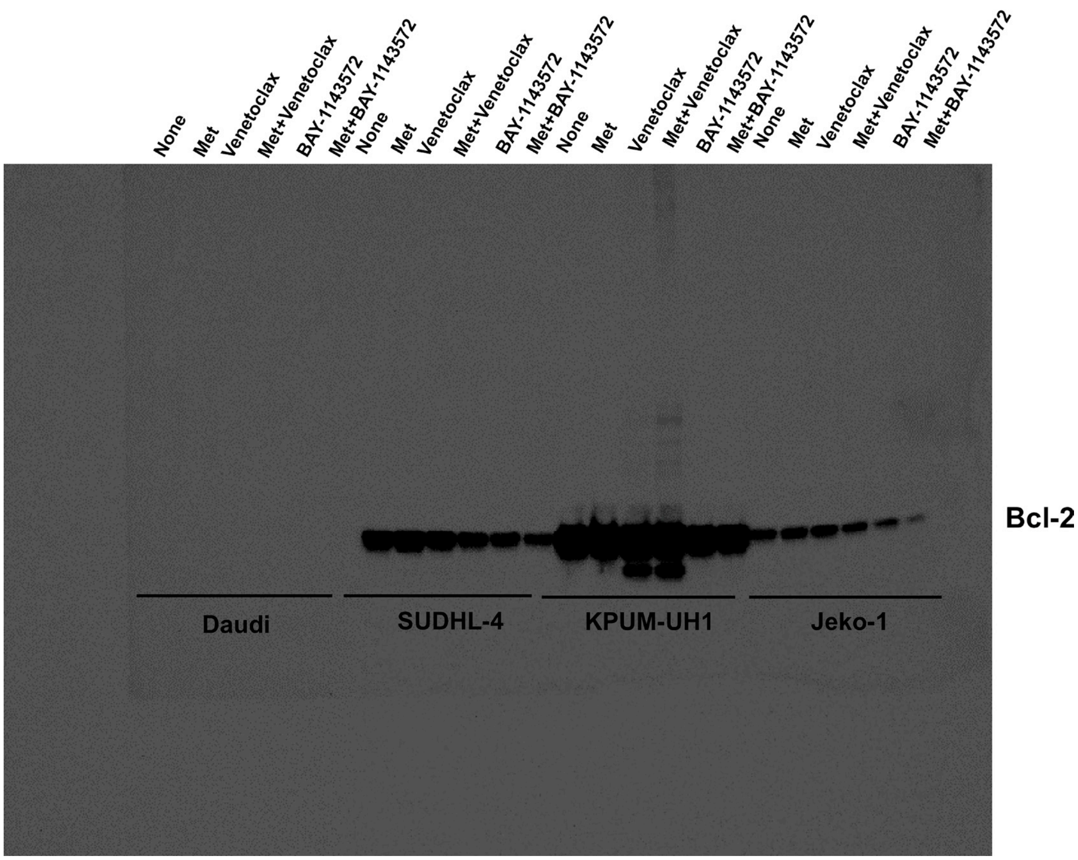

Supplementary Figure 5: Contrasted whole gel image of bcl-2 image shown in Figure 6.
